# In Vivo Anti-Malarial Activity of *Heracleum persicum* Fruit Extract, in Combination with Chloroquine against Chloroquine–Sensitive Strain of *Plasmodium berghei*

**Published:** 2018-06

**Authors:** Nahideh MAZHARI, Mehdi NATEGHPOUR, Peyman HEYDARIAN, Leila FARIVAR, Effat SOURI, Afsaneh MOTEVALLI HAGHI

**Affiliations:** 1. Students’ Scientific Research Center, Tehran University of Medical Sciences, Tehran, Iran; 2. Dept. of Medical Parasitology and Mycology, School of Public Health, Tehran University of Medical Sciences, Tehran, Iran; 3. Center for Research of Endemic Parasites of Iran, Tehran University of Medical Sciences, Tehran, Iran; 4. Dept. of Medical Parasitology and Mycology, School of Medicine, Qazvin University of Medical Sciences, Qazvin, Iran; 5. Dept. of Pharmaceutical Chemical, School of Pharmacy, Tehran University of Medical Sciences, Tehran, Iran

**Keywords:** *Heracleum persicum*, *Plasmodium berghei*, Chloroquine, Combination, In vivo

## Abstract

**Background:**

We evaluated the anti-malarial activity of *Heracleum persicum* individually and in combination with chloroquine.

**Methods:**

This study was conducted at the School of Public Health, Tehran University of Medical Sciences, Tehran, Iran in 2015–2016. The Peter’s method was used for determining fifty percent effective dose (ED50) of the *H. persicum* extract and chloroquine individually against chloroquine sensitive *P. berghei* in small white mice. Six experimental groups for *H. persicum* and 6 groups for chloroquine and two control group (positive and negative) were considered for determination of ED50. Interaction between *H. persicum* and chloroquine also was evaluated based on fixed ratios method. Ratios of 0/100, 20/80, 40/60, 60/40, 80/20, 100/0 of ED50 of chloroquine and *H. persicum* respectively were tested against the parasite. Then inhibitory effects of two drugs were calculated and plotted in the relevant graphs.

**Results:**

Overall, 1500 mg/kg, and 1000 mg/kg concentrations of *H. persicum* against *P. berghei* resulted in ED50 and ED74 respectively. ED50 of chloroquine against the parasite was obtained as 1.4 mg/kg of mouse body weight. Moreover, combination of *H. persicum* and chloroquine showed a weak potentiation in ratios of 40/60 (chloroquine +*H. persicum*) with 64% inhibition, but not in other ratios.

**Conclusion:**

Although *H. persicum* individually showed a reasonable antimalarial efficacy against chloroquine sensitive *P. berghei*, in combination with chloroquine it showed additive or antagonism result except in ratios of 40%CQ+60%HP.

## Introduction

Malaria is a parasitic disease that remains one of the most problems in public health in the developing countries. Five species of *Plasmodium* genus cause the disease in human including *P. falciparum* agent of malignant and severe malaria (1). Malaria affects mostly population living in tropical and subtropical countries in Africa, Asia, Latin America and islands in the South-West Pacific (2). It caused 438000 deaths in 2015, with the most burden in children under 5 yr of age in Africa. According to WHO programme for malaria elimination 57 countries were on track to achieve the Health Assembly’s target of reducing their malaria burden about 75% by 2015 in malarious areas (3). At present, Sistan and Baluchistan and Hormozgan provinces are endemic areas in Iran. Although national malaria elimination programme is in process in Iran, due to traffic of Afghani and Pakistani immigrants from southeastern borders into the country, some cases are reported annually in Iran (4, 5). The prevalence of antimalarial resistant *P. falciparum* strains in the world and recently in *P. vivax* in some malarious areas has resulted in an increased risk of the disease (6).

Malaria management, including early diagnosis and effective treatment, remains a fundamental part of malaria control and eradication strategies (3). Based on WHO guidelines, combination therapy in uncomplicated falciparum malaria would be implemented with Artemisinin-based combination as the first line treatment of the infection (7, 8). Different species of medicinal plants have been introduced to treat malaria and other febrile diseases. Poverty in the endemic areas is the most problem for accessing the poor people to effective medicines, therefore, there is high tendency of them to use the traditional herbal remedies particularly for treatment of malaria in these regions (9).

There are about 500000 plant species in the world that only 1% of them have been investigated in the field of pharmaceutical studies. Since geographical conditions, more variety of the medicinal plant species can be found in Iran than European countries (10). *Heracleum persicum*, commonly known as Golpar (feather flower) or Persian Hogweed, is a flowering, perennial plant in the family of Apiaceae. It grows in wetlands, riverbanks and humid mountainous regions in Iran. Its fruits are widely used as spices, preparation of pickles in Iranian nutrition and as carminative in folk medicine (11). Golpar is one of the 51 plants used to treat Tabe Reba (malaria like fever) in Iranian traditional medicine (12). This plant has antioxidant, anticonvulsant, analgesic, anti-inflammatory, antiseptic, anti-helminths and immunomodulatory activities (11, 23). Fruit extract of *H. persicum* is composed of terpenoids, monoterpenes, isoterpenoid, pimpinellin, isopimpinellin, bergapten, isobergapten and spending (11, 13). Many surveys have been reported on the anti-malarial activity of terpenes (14–16).

Since terpenoids and monoterpenes are the main constituents of *H. persicum* extract and there is not more scientific data to validate anti-malarial activity of this medicinal plant, this study was conducted to evaluate anti-malarial activity of this plant individually and in combination with chloroquine, against chloroquine – sensitive strain of *P. berghei*.

## Materials and Methods

### Herbal extract and drugs

This study was conducted at the School of Public Health, Tehran University of Medical Sciences, Tehran, Iran in 2015–2016. Plant materials were collected from Mazandaran Province, northern Iran and were identified with Department of Pharmaceutical Chemical, Tehran University of Medical Sciences, Iran. Five hundred grams of air-dried fruits were ground and macerated in ethanol at room temperature and then extracted using an extractor. After evaporating the excess ethanol the resultant crude extracts were stored at low temperature (4 °C) until use in anti-malarial assays. Several concentrations of the extract as 500, 1000, 1250, 1500, 1750 and 2000 mg/kg were prepared in sterile normal saline containing 2.5% Tween 20. For control group, only normal saline was used as placebo. Chloroquine (Sigma Chemical) was administered in concentrations of 0.5, 1, 1.5, 2, 4 and 8 mg/kg.

### Parasite

Chloroquine-sensitive NICD strain of *P. berghei* was injected into donor mice by intraperitoneal inoculation. The infected blood was collected via cardiac puncture and diluted in normal saline in such a way that, 0.2 ml of the blood contained about 1×10^6^ infected red cells.

### Animals

Small white male mice with 20 ± 2 gr weights were used in this study. They were kept in plastic cages at room temperature and were fed with standard diet.

For the experimental processes on the mice, a medical ethics was obtained from Ethical Committee of Tehran University of Medical Sciences under the register number of ir.tums.rec.1394.1813.

### Antiplasmodial Assays

Antiplasmodial assays were first carried out to determine ED50 (fifty percent of effective dose) of the individual drugs (*H. persicum* and chloroquine) according to 4-d suppressive test (17). At the first step, for each previously mentioned concentrations of chloroquine (0.5, 1, 1.5, 2, 4 and 8 mg/kg) and *H. persicum* (500, 1000, 1250, 1500, 1750 and 2000 mg/kg) 5 mice were considered as experimental groups. Furthermore, two other groups considered as control negative and positive. All of groups were infected with 10^6^ *P. berghei* parasitized erythrocytes via intraperitoneal. The first dose of the extract concentrations and chloroquine were administered subcutaneously 2 h after injection of the infected erythrocytes on day 0 and then administration of the doses was continued daily up to day 3. Positive control group received CQ (20 mg/kg) at the same process. Five untreated mice were allocated to the negative control group. Thin blood smears were prepared on days 4, 7, 14 and 21 from tail blood and stained with 3–5% Giemsa (18). The parasitemia of each mouse was counted against 10000 erythrocytes. The ED50 value of the chloroquine and extract calculated using following formula:

$$\%\;\mathrm{Inhibition}=\frac{\mathrm{mean}\;\mathrm{parasitemia}\;\mathrm{of}\;\mathrm{control}\;\mathrm{group}-\mathrm{mean}\;\mathrm{parasitemia}\;\mathrm{of}\;\mathrm{treated}\;\mathrm{group}}{\mathrm{mean}\;\mathrm{parasitemia}\;\mathrm{of}\;\mathrm{control}\;\mathrm{group}}$$

### Combination treatment

Obtained ED50s for both chloroquine and *H. persicum* were used for combination therapy. Combination of two mentioned drug solutions was prepared in fixed ratios of 100/0, 80/20, 60/40, 40/60, 20/80 and 0/100 of HP/CQ according to fixed ratios technique (19). Briefly, the infected mice were exposed to prepared ratios until day 3. The method of test for this step was the same to that described for ED50 determination. Parasitemia was calculated in thin blood smears on days 4, 7 and 14. Interaction between chloroquine and *H. persicum* against the parasite was interpreted as lying the points above the joint line indicated to synergism and either around the line or below indicated to additive or antagonism respectively. The mean survival time for each group was recorded during the study.

### Statistical analysis

Percentage inhibition and survival time during study were presented as mean ± standard deviation for all groups. The mean percentage parasitemia on day 4, 7 and 14 and the mean survival time were analyzed statistically using ANOVA test to identify the significant differences between test groups and negative control group using *P*-value criteria (<0.05).

## Results

ED50s inhibitory effect of ethanolic extract of the plant and chloroquine were obtained 1500mg/kg and 1.4mg/kg respectively (Table 1). Concentration of 1000 mg/kg of *H. persicum* was found to be more effective with 74% inhibition of parasitemia growth in comparison with the others (Table 2). The results of antimalarial activity of *H. persicum* showed that there was a significant difference between the means of parasitemia at various concentrations when compared with the negative control group (*P*<0.05)

**Table 1:** ED50 concentration of *Heracleum persicum* and chloroquine against the chloroquine-sensitive strain of *P.berghei*

***Parasite***	***Drugs***			
	***H. persicum***		***Chloroquine***	
	***Dose of ED50(mg/kg)***	***Parasitemia inhibition(%)***	***Dose of ED50(mg/kg)***	***Parasitemia inhibition(%)***
Chloroquine-sensitive *P. berghei*	1500	48±9.8	1.4	48.3±7.6

**Table 2:** Antimalarial effect of *Heracleum persicum* against the chloroquine sensitive-strain of *P.berghei* on day 4

***Groups***	***Dose(mg/kg)***	***parasitemia Percent (Mean±SD)***	***Parasitemia inhibition (%)***
*H. persicum*	500	14.45±1.1	46
*H. persicum*	1000	6.89±1.7	76
*H. persicum*	1250	11.7±1.8	57.6
*H. persicum*	1500	13.1±2.5	50
*H. persicum*	1750	10.65±0.6	61.5
*H. persicum*	2000	10.55±1.33	61.5
Without treatment	-	26±5.07	-
Chloroquine	20	0.0	100

**Fig. 1:**
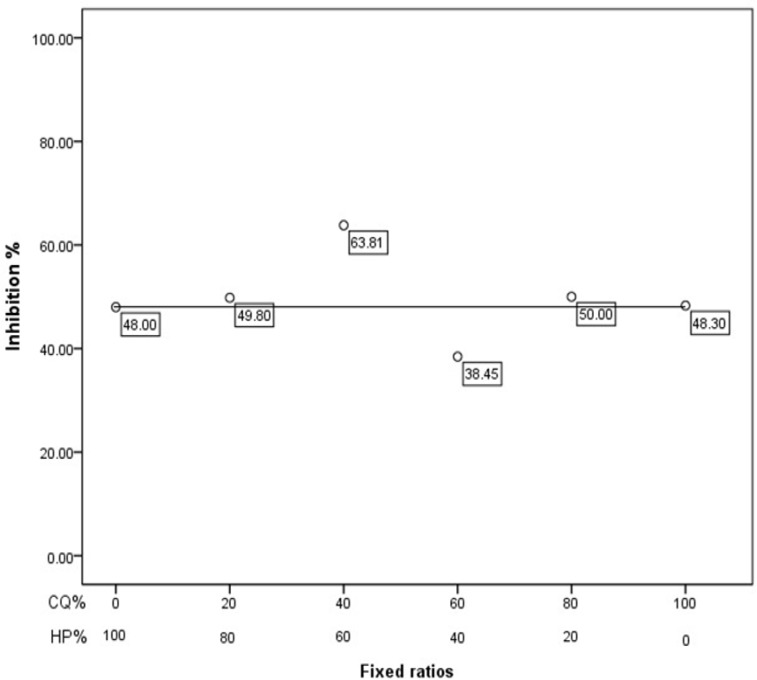
Interaction between *Heracleum Persicum* and chloroquine against the chloroquine-sensitive strain of *P. berghei*

Results of the activity of the antiplasmodial drug in various combinations have been shown in Table 3. The ratio of 60/40 (*H. persicum*/chloroquine) had the highest activity with 64% parasite suppression that indicates a synergism efficacy (Fig. 1)

**Table 3:** Fixed ratios of *Heracleum persicum* and chloroquine combination and their relevant effective doses on the chloroquine sensitive-strain of *P. berghei*

***Fixed ratios CQ/HP***	***Parasitemia inhibition% (Mean±SD)***
0/100	48±9.8
20/80	49.8±15.1
40/60	63.81±7.3
60/40	38.45±11.2
80/20	50±8.4
100/0	48.3±7.6

**Fig. 2:**
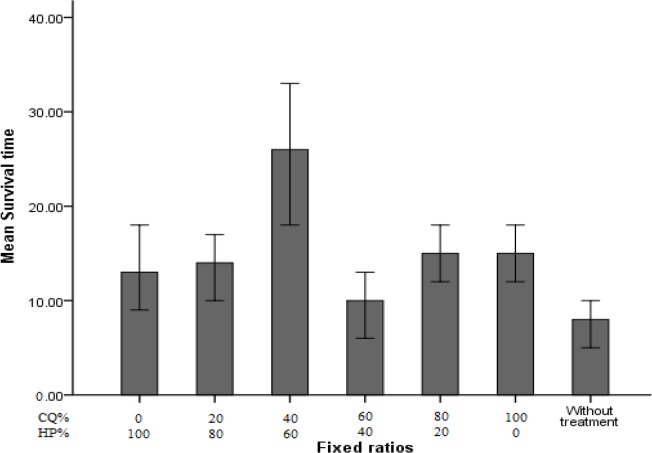
Mean survival time in infected mice treated with different ratios of chloroquine and *Heracleum persicum* against the chloroquine-sensitive strain of *P. berghei*

The curve obtained by other combinations of *H. persicum* with chloroquine is evidence of an antagonistic or additive interaction. As it was expected the survival time of treated mice with ratio of 60/40 between *H. persicum* and chloroquine (26.4 d) was longer than other groups throughout the 28 d follow up (Fig. 2). Moreover, ANOVA results revealed significant difference in mentioned ratio than other groups (*P*<0.001).

## Discussion

Medicinal plants are still widely used in the treatment of malaria and other diseases. Terpenes are the main constituents of medicinal plants (such as *Artemisia* and *Cassia*) which have various therapeutic properties, justifying their use in traditional medicine (20–22). There are some reports in the field of antimalarial activity of terpenes (14–16). For instance results of antimalarial activity of *A. annua*, a terpenoid-rich species showed that the concentrations of 1100 and 1300 mg/kg caused a significant reduction in parasitaemia (7). Despite presence of terpenoids and monoterpenes in *H. persicum* constituents and its using in traditional medicine, we did not find any documented information about its antimalarial activity (10, 15).

We tried to examine the effectiveness of *H. persicum* fruit extract against malaria parasite of *P. berghei*. A survey of *H. persicum* for antimicrobial activity indicated that leaf extracts of the plant inhibited the growth of *Acinetobacter baumannii*. This finding is in agreement with study that exhibited extracts from the roots and aerial parts of the plant completely inhibited the growth of *Bacillus anthracis* (23). The effects of ethanol extract of *H. glabrescens* was investigated on *Giardia* cysts. Concentration of 200 mg/ml for 60 min, had 44% fatal effect (24). In vector control study *H. persicum* essential oil effectiveness as a larvicidal substance was evaluated against *Anopheles stephensi* in southern Iran. About 50% and 90% of the larvae were killed with exposing to 26.30 and 114.40 ppm of the oil respectively (25).

Effectiveness of the aqueous extract of *H. persicum* has examined against mice peritoneal macrophages that resulted in considerable effect on increase of nitric oxide production in 10mg/ml of the extract. Moreover, an increase was observed in ROS and candidacidal activities of macrophages when exposed to concentration of 20 mg/ml of the extract (26). Another study exhibited anti-inflammatory property of *H. persicum* extract. Indeed, functional mechanisms of plant-derived terpenoid components modulate the nuclear factor-kB (NF-kB) signaling, that have major role in the pathogenesis of inflammatory diseases (27). Present study considered the scientific reasons behind the folkloric use of *H. persicum* in the treatment of Tabe Reba (malaria like fever) in Iranian traditional medicine and inhibitory activity of *H. persicum* was evaluated on chloroquine sensitive strain of *P. berghei*. The inhibition of 63% to 74% in different concentrations and the highest inhibition occurred in the concentration of 1000 mg/kg with inhibitory effect of 74%. Moreover in combination treatment ratio of 40/60 (chloroquine +*H. persicum*) was more effective than the others resulted in 64% inhibition.

## Conclusion

*H. persicum* has, more or less, antimalarial efficacy and may explain the reason of administrating the herb in malaria like fever treatment claimed by traditional healers. Therefore, the plant is worth interested as an antimalarial agent developed into phytodrugs.

## Ethical considerations

Ethical issues (Including plagiarism, informed consent, misconduct, data fabrication and/or falsification, double publication and/or submission, redundancy, etc.) have been completely observed by the authors.
